# Seroprevalence and risk factors of Chikungunya in Ethiopia: a systematic review and meta-analysis

**DOI:** 10.3389/fpubh.2025.1538911

**Published:** 2025-03-11

**Authors:** Gashaw Getaneh Dagnaw, Abebe Tesfaye Gessese, Solomon Lulie Abey, Abebe Belete Bitew, Kassahun Berrie, Haileyesus Dejene

**Affiliations:** ^1^Department of Veterinary Biomedical Science, College of Veterinary Medicine and Animal Sciences, University of Gondar, Gondar, Ethiopia; ^2^Department of Veterinary Pathobiology, College of Veterinary Medicine and Animal Sciences, University of Gondar, Gondar, Ethiopia; ^3^Department of Veterinary Epidemiology and Public Health, College of Veterinary Medicine and Animal Sciences, University of Gondar, Gondar, Ethiopia

**Keywords:** Chikungunya, Ethiopia, meta-analysis and systematic review, risk factors, seroprevalence

## Abstract

**Introduction:**

The resurgence of the Chikungunya virus has led to public health concerns due to frequent epidemics worldwide. Chikungunya was first detected in Ethiopia in 2016, and it has been identified in various regions. However, the current status of the disease in Ethiopia remains unknown, underscoring the need for updated information.

**Objective:**

To provide up-to-date epidemiological data on the status of Chikungunya in Ethiopia.

**Methods:**

A systematic review and meta-analysis were conducted using the PubMed, Scopus, and Google Scholar databases in accordance with the PRISMA guidelines, the literature search was conducted from September to October 2024. The search terms used included ‘Chikungunya,’ ‘Chikungunya Virus,’ ‘Prevalence,’ ‘Seroprevalence,’ ‘Risk Factor,’ and ‘Ethiopia.’ The inclusion criteria covered online articles published between 2016 and 2024 in English and published in Ethiopia. The quality assessment involved independent expert evaluations, and publication bias was assessed using Begg’s and Egger’s tests. The analysis was performed using STATA 17 software.

**Results:**

A total of five articles met the eligibility criteria and were included in the data extraction. The pooled seroprevalence of Chikungunya in Ethiopia was 24.0%. The highest seroprevalence was reported in the Southern Nations, Nationalities, and Peoples’ Region (SNNPR), at 43.6%, while the lowest seroprevalence was in Dire Dawa, at approximately 12.0%. Factors such as occupation, education, age, and sex contributed to the variation in seroprevalence of the disease. Subgroup meta-analysis revealed heterogeneity across the types of studies included. No indications of publication bias or small-study effects were found according to Begg’s test or Egger’s test.

**Conclusion and relevance:**

The pooled prevalence of Chikungunya underscores its significance in Ethiopia, necessitating proactive monitoring, active viral disease surveillance, and robust health system enforcement.

## Highlights

- Chikungunya is a neglected tropical disease.
- The Chikungunya virus was first detected in Ethiopia in 2016.
- The pooled seroprevalence of Chikungunya in Ethiopia is 24.0%.
- The seroprevalence of Chikungunya varies with different factors.
- Seroprevalence is higher in males and farmers.
- ELISA is commonly used for detecting the virus in Ethiopia.

## 1 Introduction

Chikungunya, a mosquito-borne viral disease, is caused by an RNA virus belonging to the Alphavirus genus of the family Togaviridae known as Chikungunya virus (CHIKV). It is responsible for millions of documented cases worldwide (1). The disease is characterized by clinical signs such as fever, debilitating severe joint pain, joint swelling, muscle pain, headache, nausea, fatigue and rash (2).

Chikungunya virus was first isolated during a 1952–53 outbreak in southern Tanzania (3), although clinical descriptions suggest its presence as far back as the 1600S (4). Today, CHIKV has become widespread globally, has been identified in more than 110 countries and represents a significant global public health concern (5). Factors such as climate change, vector adaptations, urbanization, and human migration have contributed to the spread of the virus to new areas (6). In addition, studies have shown that the incidence of Chikungunya varies with factors such as occupation, age, sex, and education. Farmers and older people are associated with higher prevalence rates. Gender disparities also play a role in influencing exposure and transmission patterns, as the occurrence of the disease is higher in female (7–12).

Chikungunya in Ethiopia is becoming a significant public health concern, as it has caused considerable morbidity since it was first detected (13). The virus was first documented in Ethiopia in June 2016, with the confirmation of its first case in the Suuf kebele, Dollo Ado district of the Somalia regional state (14), which is assumed to have originated from Kenya. The Somalia regional state shares a border with the Mandera region of Kenya, where a Chikungunya outbreak was ongoing (15). Since then, Chikungunya has spread rapidly and has been reported in different districts of Ethiopia (16, 17).

At present, there are no approved vaccines or antiviral therapies available for Chikungunya (18). Meanwhile, nucleic acid therapeutics are emerging as transformative agents in antiviral treatment, leveraging precise genetic interventions to combat viral infections (19). However, the main approach for treating and controlling and preventing this disease is through alleviating symptoms or supportive treatment and eliminating the mosquitoes that transmit the virus (18, 20). In addition, public awareness creation through community education and training about the outbreak of emerging and reemerging vector-borne diseases, methods of transmission, and control and prevention methods remain important mechanisms for managing Chikungunya (18).

Like many developing nations, Ethiopia cases involve struggles with a range of public health challenges that contribute to the outbreak of disease. Limited healthcare infrastructure and uneven distribution of resources hinder effective prevention, detection, and response to health crises (21). Despite these limitations, Ethiopia has made an effort to limit the spread of CHIKV across the country and to prevent the potential transmission of the disease in affected regions (13). The government was taking vector control measures such as indoor residual spraying, distributing insecticide-treated bed nets and encouraging the population to eliminate a breeding ground for mosquitoes such as stagnant water (22). However, the effectiveness of these measures may vary, and there are reports of the disease from various locations in Ethiopia. The disease’s current status in Ethiopia is unclear. Knowing the current status of the disease in the country is important. The outcome provides insights for health professionals and concerned authorities to develop effective control and prevention strategies. Therefore, this systematic review and meta-analysis aimed to provide up-to-date epidemiological data on the status of Chikungunya in Ethiopia.

## 2 Methods

### 2.1 Systematic review protocols

**Figure 1:**
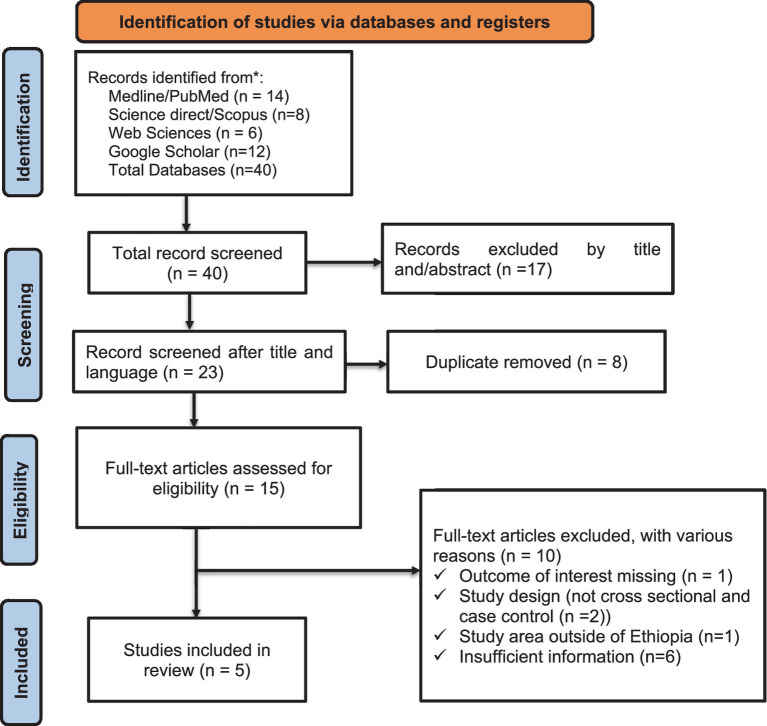
PRISMA flowchart for the selection of studies on the CHIKV in Ethiopia.

The guidelines and procedures of the Preferred Reporting Items for Systematic Reviews and Meta-Analysis (PRISMA) (23) were followed in this systematic review and meta-analysis (Figure 1) and registered in the database of the Prospective Register of Systematic Reviews (PROSPERO) under the reference number CRD42023271579.

### 2.2 Literature search strategy

The data were extracted from published public articles available from different electronic databases, including Medline/PubMed, Science Direct/Scopus, Google Scholar and Web of Science. The literature search was conducted from September to October 2024, applying language restrictions to English published between 2016 and 2024. Mendeley version 1.19.8 (Mendeley Ltd) was used for search, collection and removal of duplicates of articles. A set of keywords, such as “Chikungunya,” “Chikungunya Virus,” “Chikungunya Fever,” “Vector-borne,” “Arbovirus,” “Incidence,” “Prevalence,” “Seroprevalence,” “Seroepidemiology,” “Risk Factors,” “Potential Factors” and “Ethiopia,” were used in the search. The search queries were configured using Medical Subject Headings (MeSH), and the “OR and “AND” Boolean operators were used to identify studies with any of the keywords in their titles, abstracts and full texts that might be included in this systematic and meta-analysis review. Moreover, unpublished thesis manuscripts were also accessed from various Ethiopian universities and research centers.

### 2.3 Inclusion and exclusion criteria

Regardless of the research population type, articles reporting CHIKV infection or disease conducted in Ethiopia and involving the general population or a specific age group were included in this study. The study’s objectives served as the basis for defining the inclusion and exclusion criteria. The inclusion criteria include articles that focused on CHIKV infection or disease, written in English, published between 2016 and 2024, using case–control, cross-sectional and cohort studies in which the seroprevalence of Chikungunya was confirmed via laboratory tests, encompassing serologic or virological antibody detection methods such as ELISA IgG, ELISA IgG + IgM and/or molecular diagnosis, and full-text articles included in the systematic review and meta-analysis. Papers are rejected based on the exclusion criteria. These criteria include articles that were unpublished, systematic reviews and meta-analysis, did not align with the specified emphasis outlined in the systematic review, exclude important things about seroprevalence, not studied in Ethiopia and not written in English. The analysis did not take into account the specificity or sensitivity of the tests.

### 2.4 Data extraction

Two experts (ATG and SLA) independently identified articles from the search engines using key terminologies and subsequently screened them based on their titles and abstracts. The selected publications were then imported into Mendeley, after which the full texts were retrieved. The eligibility of the full texts was assessed by checking whether they addressed the main outcomes of interest. The proportion of seropositive Chikungunya individuals was considered the main outcome of the study. Data extraction took place independently by two experts (GGD) from November 1, 2024, to November 21, 2024, and cross-verification was performed by another two experts (ATG & HD). If discrepancies arose, the data were re-extracted, even if there were no initial discrepancies.

### 2.5 Quality and risk-of-bias assessment

A comprehensive search for all possibly relevant articles and the application of precise, repeatable criteria for article selection were two of the tactics we used to reduce bias and random error. An established systematic methodology that complies with evidence-based methodological standards was followed in evaluating research designs and study characteristics, the synthesis of data, and the interpretation of the findings. To choose which papers to include and omit from the review, HD and GGD examined the titles, abstracts, and full-text publications. After that, the articles are evaluated to see if they meet the specified eligibility requirements. BK assessed quality using the appraisal tool for cross-sectional studies (AXIS tool) (Supplementary File 1). There are 20 items on this checklist (24, 25). In addition, the presence of publication bias or small-study effects was assessed by using Begg’s test and regression-based Egger test, which examine the correlation between the effect size and the standard error of the effect size across studies (26).

### 2.6 Data synthesis and meta-analysis

The extracted data were entered into an Excel spreadsheet (2019). The seroprevalence of Chikungunya from each study was recorded, and the individual study weight, standard error, and 95% confidence interval (CI) were calculated based on the inverse variance method and the binomial equation (27). The logit transformation of the proportional prevalence with its variance and standard error was calculated. Subgroup analyses for the primary outcome (seroprevalence of Chikungunya) were performed using the DerSimonian and Laird model by considering geographical locations and laboratory techniques employed (PCR or ELISA) (28). The heterogeneity among and within the studies was estimated from the inverse variance of the random effects model (29). The parameters tau-squared (τ^2^), I-squared (I^2^) and H-squared (H^2^) were calculated to measure the variance in the true effect sizes between-study variance, interstudy heterogeneity, and total variability, respectively (30). The pooled prevalence and standard error with 95% confidence intervals (CIs) were calculated (31). STATA version 17 software was used to perform the statistical tasks. A *p*-value less than 0.05 (*p* < 0.05) was considered significant in all analyses (32).

## 3 Results

### 3.1 Literature search results

This systematic review and meta-analysis focused on published studies regarding Chikungunya in Ethiopia. A total of 40 articles published between 2016 and 2024 were identified using search engines. Of these, 17 articles were rejected based on their titles and abstracts, which indicated irrelevance to our review. The remaining 23 studies underwent further evaluation, resulting in the exclusion of eight duplicates or inappropriate articles. A total of 15 full-text papers were accessed and assessed for eligibility using pre-set criteria, leading to the inclusion of five studies in the systematic review and meta-analysis. The remaining 10 articles were excluded based on study area and other factors. Ultimately, five relevant studies were included in this systematic review and meta-analysis (Figure 1).

### 3.2 Study characteristics

**Figure 2:**
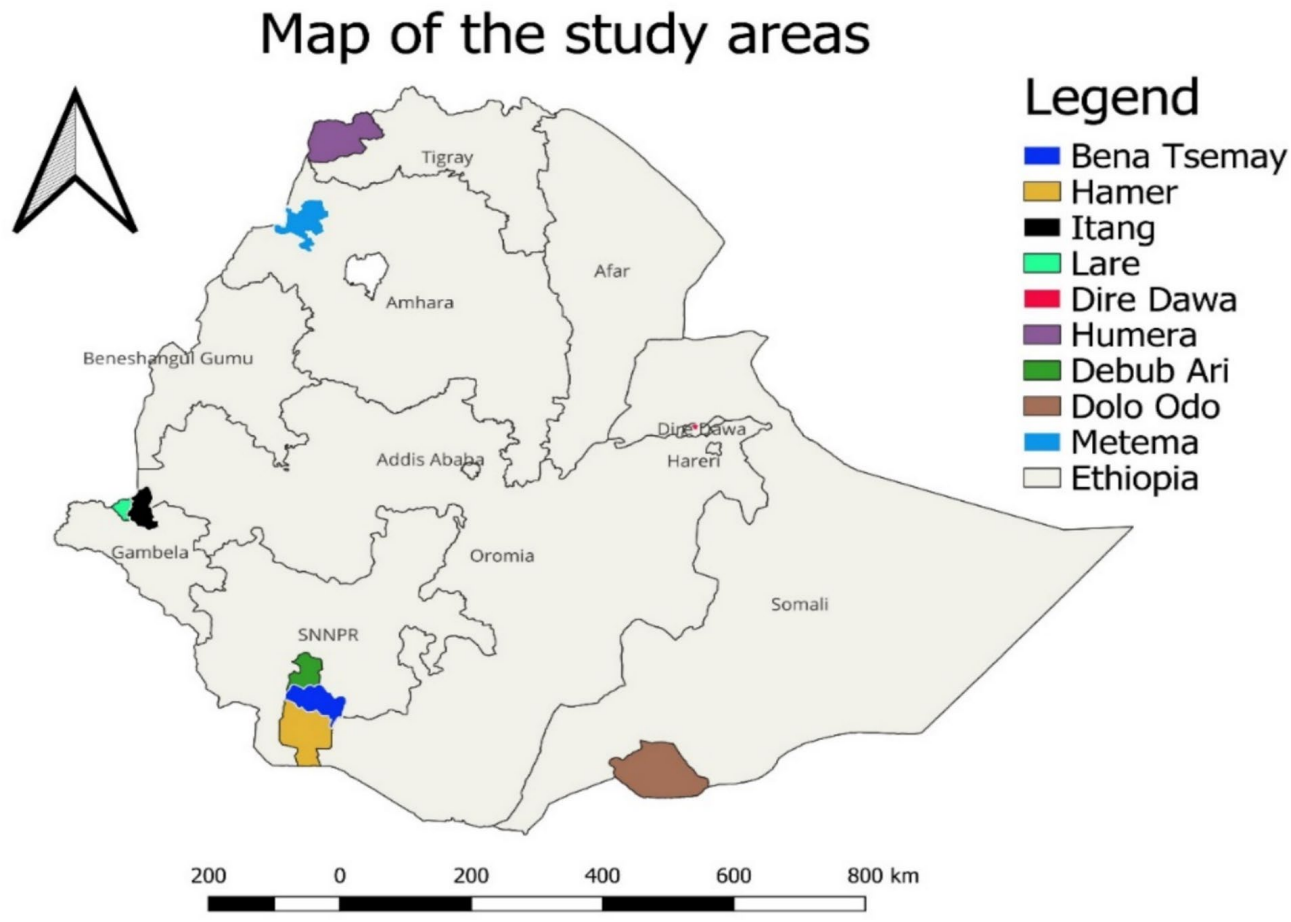
Map of Ethiopia showing Chikungunya report (Drawn by QGIS 3.34.1).

The selected articles encompassed research conducted in five regional states—South Nations and Nationalities Region, Amhara, Tigray, Gambella, and Southeast Ethiopia—as well as one city administration (Dire Dawa) (Figure 2). The included studies were cross-sectional (80%, 4/5) and one case–control study (20%, 1/5). There were two types of assays used for diagnosis across the studies, namely, the enzyme-linked immunosorbent assay (ELISA; 80%, 4/5) and quantitative reverse transcription polymerase chain reaction (RT–qPCR; 20%, 1/5) (Table 1).

**Table 1:** Seroprevalence of Chikungunya virus in Ethiopia from selected publications between 2016 and 2020.

References	Study area		Study design	Sample size	Test type	Sero-prevalence (%)	Logit (95% CI)
	Region	District					
Endale et al. (33)	SNNPR	Debub Ari	Cross-sectional	190	ELISA	51.58	0.15 (−0.22, 0.35)
	SNNPR	BenaTsemay		35	ELISA	25.71	0.39 (−1.82, −0.30)
	SNNPR	Hamer		135	ELISA	37.04	0.18 (−0.88, −0.18)
Ferede et al. (34)	Amhara	Metema	Cross-sectional	274	ELISA	30.66	0.13 (−1.07, −0.56)
	Tigray	Humera		312	ELISA	16.35	0.15 (−1.93, −1.33)
Asebe et al. (35)	Gambella	Itang	Cross-sectional	58	ELISA	20.69	0.32 (−1.98, −0.71)
	Gambella	Lare		32	ELISA	6.25	0.73 (−4.14, −1.28)
Takele (36)	South–Eastern Ethiopia	Dolo ado	Case–control	99	RT–qPCR	14.14	0.29 (−2.37, −1.24)
Geleta et al. (17)	Dire Dawa	Dire Dawa	Cross-sectional	334686	RT–qPCR	12.30	0.01 (−1.97, −1.95)

CI, Confidence interval.

### 3.3 Seroprevalence of Chikungunya

The pooled seroprevalence of Chikungunya in Ethiopia was 12.4% (95% CI: 12.24, 12.46%). The highest prevalence was reported in the Southern Nations, Nationalities, and Peoples’ Region (SNNPR) at 43.6%, while the lowest seroprevalence was found in Dire Dawa, at approximately 12%. At the district level, the highest prevalence of Chikungunya infection occurred in the Bebub Ari district (51.58%; SE (logit) = 0.15, 95% CI = −0.22, 0.35), while the lowest prevalence was recorded in the Lare district (6.25%; SE (logit) = 0.73, 95% CI = −4.14, −1.28) (Table 1).

### 3.4 Meta-analysis

**Figure 3:**
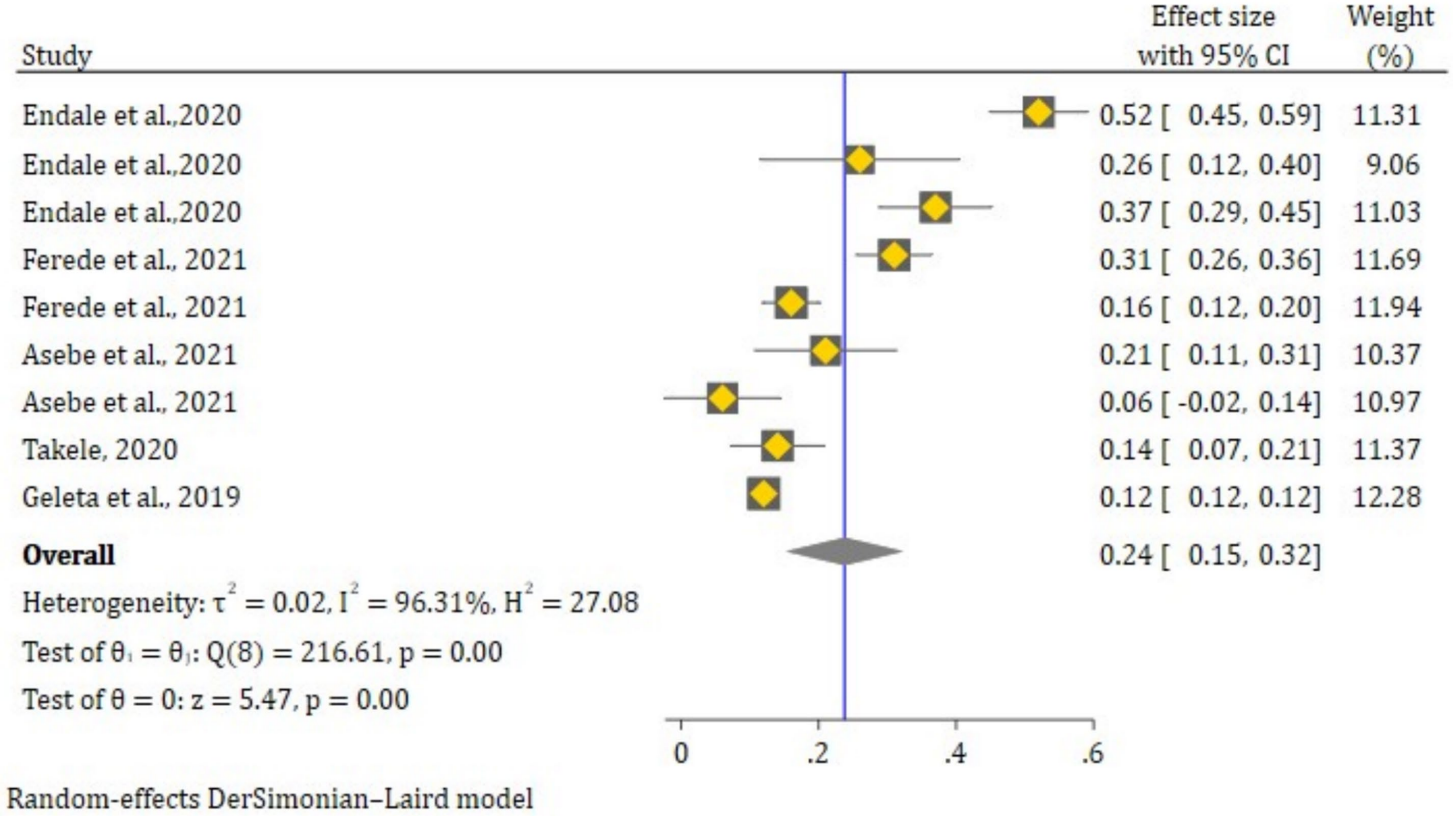
The random effect estimates of the effect size in different districts of Ethiopia.

The overall pooled effect size of Chikungunya was 24.0% (95% CI: 15.0, 32.0%) across all eligible studies. There was significant heterogeneity in the reports of Chikungunya seroprevalence between studies (*tau^2^* = 0.02, *H^2^*, 27.8, *I*^2^ = 96.35%, Q-test = 216.6, df = 8, *p* ≤ 0.001) (Figure 3).

### 3.5 Quality assessment result

In this review, a range of studies, with quality ratings from low to moderate, was evaluated. In the current meta-analysis, eight articles used the random sampling method. Additionally, all eight studies (88.9%) obtained a sample frame from a population closely resembling the target or reference population. Of these, 9 studies (100%) met six of the 20 key criteria, including aims/objectives, definition of the target/reference population, internal consistency of results, justification of findings, sample size justification, and appropriate methodological techniques. Conflicts of interest and descriptions of statistical methods were also addressed.

### 3.6 Subgroup meta-analysis

**Figure 4:**
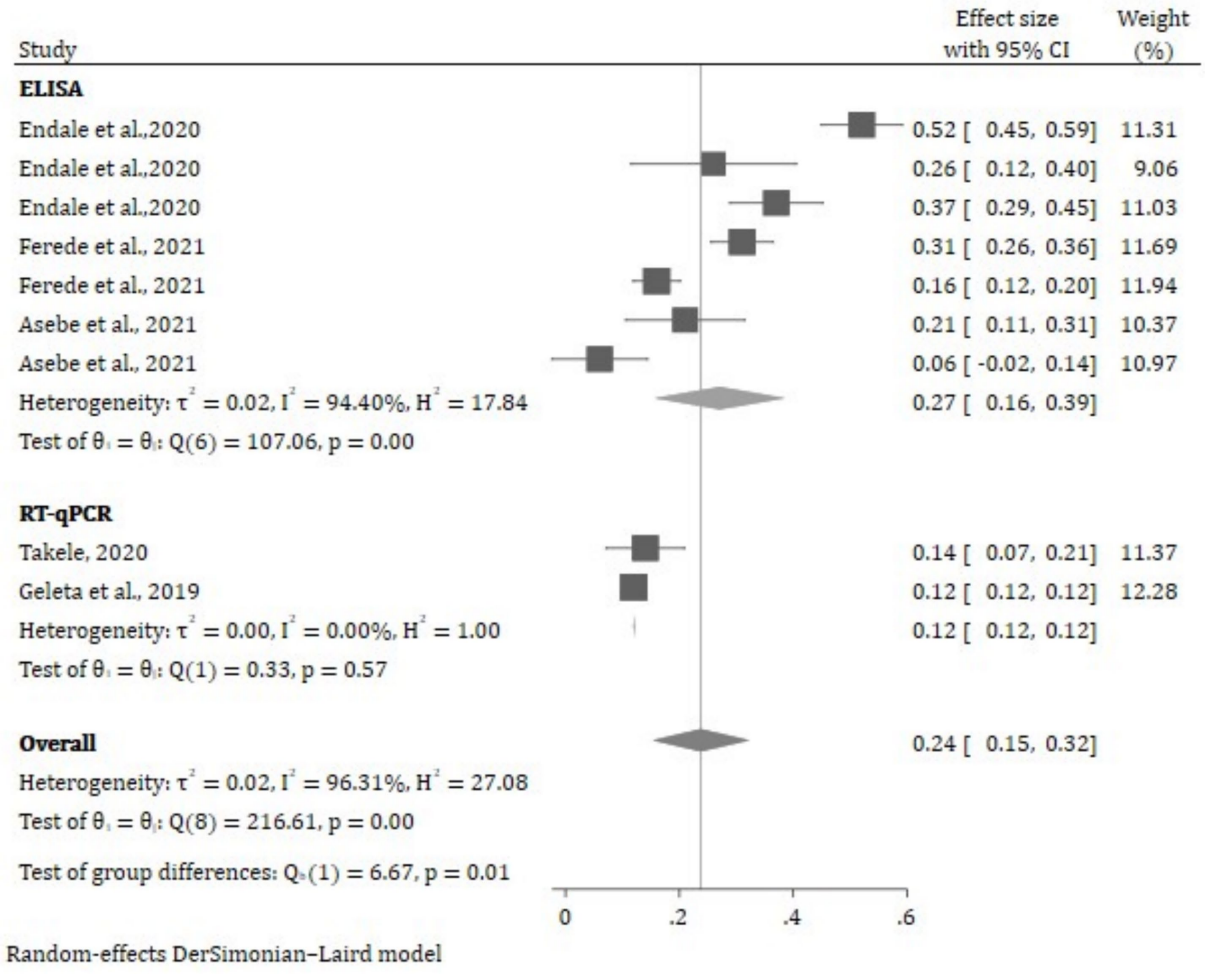
The random effect estimates of the effect size across the type of test performed.

The results of a subgroup analysis based on type of test conducted are shown in Figure 4. Accordingly, the highest pooled effect size of Chikungunya was found in ELISA technique [27.0%% (95% CI: −16.0, 39.0%)]. In addition, a high degree of heterogeneity between the various studies was shown by the subgroup analysis’s heterogeneity results (*I*^2^ = 96.31%, *p* ≤ 0.001) (Figure 4).

### 3.7 Risk factors associated with Chikungunya infection

Risk factors, including occupation, education, sex, and age, were identified in four of the included articles. The Chikungunya incidence exhibited variation based on these factors.

**Figure 5:**
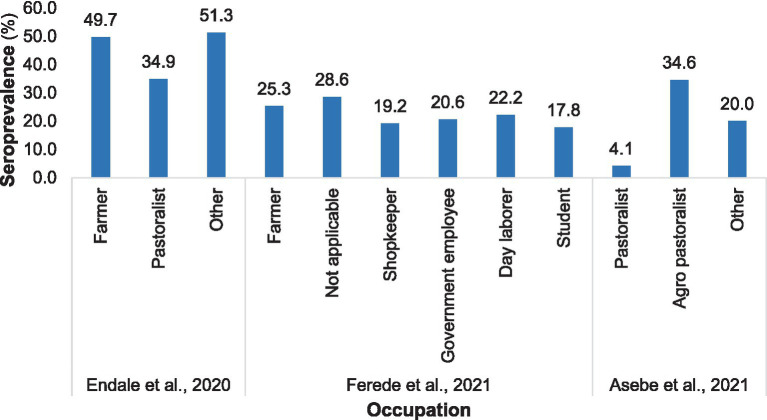
Distribution of Chikungunya among various occupations in selected publications from 2016 to 2024.

The prevalence of Chikungunya significantly varied with occupation, irrespective of geographical location. According to Endale et al. (33), farmers had a 49.7% higher seroprevalence of Chikungunya than did pastoralists (34.9%). Similar findings were reported in northwest Ethiopia by Ferede et al. (34) in 2021. Additionally, Asebe et al. (35) reported that pastoralists had the lowest Chikungunya infection rate, 4.1% (Figure 5).

**Figure 6:**
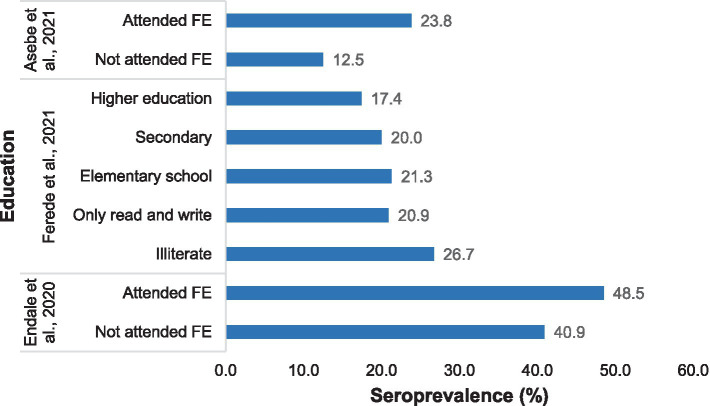
Distribution of Chikungunya according to education status in selected publications between 2016 and 2024.

The prevalence of chikungunya was greater among individuals who had received formal education than among those who had not attended formal education or were illiterate (Figure 6).

**Figure 7:**
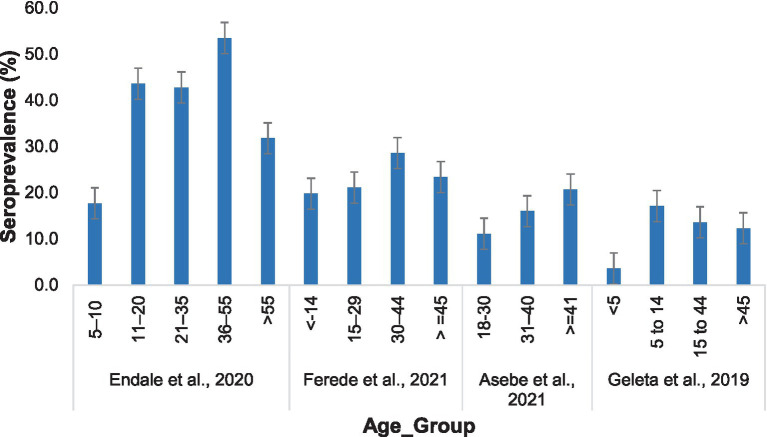
Distribution of Chikungunya among participants in different age groups in the selected publications from 2016 to 2024.

The seroprevalence of Chikungunya was greater in the adult age group, while the lowest prevalence was recorded in children. Endale et al. (33) reported that the highest Chikungunya incidence in individuals aged 36–55 years was 53.5%, and the lowest was approximately 17.7% in individuals aged 5–10 years. Similar results were reported by Ferede et al. (34). However, Geleta et al. (17) reported a higher Chikungunya infection rate in individuals aged 5–14 years (17.1%), with the lowest prevalence in those aged ≤5 years (3.6%) (Figure 7).

**Figure 8:**
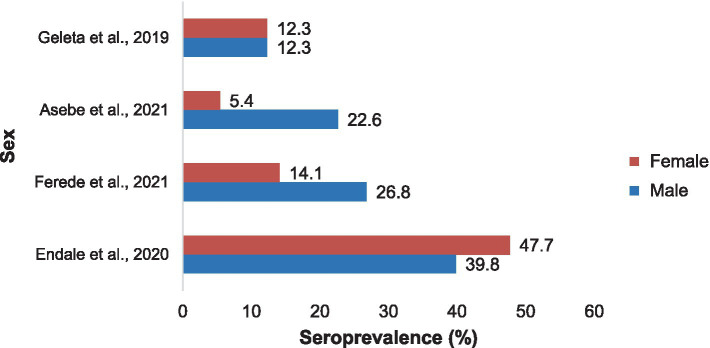
The prevalence of Chikungunya in Ethiopia in males and females in selected publications between 2016 and 2024.

Notably, the seroprevalence of Chikungunya across different sex groups was not consistent. Asebe et al. (35) reported a 22.6% higher rate of Chikungunya infection in males than in females (5.4%). Similarly, Ferede et al. (34) reported a higher prevalence of NAFLD in males (26.8%) than in females (14.1%). However, Endale et al. (33) reported a higher incidence of cancer among females (47.7%) than among males (39.8%). The approximate seroprevalence of Chikungunya in both sexes was reported by Geleta et al. (17) (Figure 8).

### 3.8 Publication bias

The statistical tests for small-study effects in a meta-analysis resulted in nonsignificant results. Begg’s test showed the absence of significant small-study effects (Kendall’s score = −8.00, SE = 9.592, *z* value = −0.94, *p* = 0.47). Similarly, the regression-based Egger test detected funnel plot asymmetry and revealed no indication of publication bias or small-study effects in the meta-analysis (beta1 = −1.74, SE of beta1 = 1.766, *z* value = −0.99, *p* = 0.32).

## 4 Discussion

Chikungunya in Ethiopia is becoming a significant public health concern, as it has caused considerable morbidity since it was first detected (13). Like many developing nations, Ethiopia struggles with a range of public health challenges that contribute to the outbreak of disease. Limited healthcare infrastructure and uneven distribution of resources hinder effective prevention, detection, and response to health crises (21). The aim of this systematic review and meta-analysis to estimate the pooled seroprevalence and identify potential risk factors of Chikungunya disease in Ethiopia.

In the current systematic review and meta-analysis five studies on Chikungunya conducted in Ethiopia and published in English between 2016 and 2024 were deemed eligible and were included in this systematic review and meta-analysis. The meta-analysis revealed that the pooled seroprevalence of Chikungunya was 24.0%. Factors such as geographical location, occupation, age, sex, and education contributed to the variation in the Chikungunya seroprevalence. Subgroup analysis based on the study area and the type of tests performed revealed significant heterogeneity.

The pooled seroprevalence of Chikungunya highlights the significance of the disease in Ethiopia. This prevalence was relatively lower than that reported in neighboring countries. For instance, Sudan seroprevalence has a median of 12% among the general population, with a range of 0–43% (8), while Kenya has prevalence rates ranging from 0.97 to 42% (15). Moreover, according to the results of this systematic review and meta-analysis, the highest seroprevalence of Chikungunya was reported in the Debub Ari district of the SNNPR, while the lowest was recorded in the Lare district of the Gambella regional state. In addition, seroprevalences differed among districts within the same region. For instance, the prevalence of Chikungunya in Itang (Gambella) was approximately triple that in Lare (Gambella). This result is in line with previous studies revealing that geographical region contributes to the inconsistency in Chikungunya incidence (37). This disparity prompted an exploration of potential contributing factors. Geographical nuances, including climate and ecosystems, may impact the abundance and behavior of Aedes mosquitoes, which are the vectors responsible for CHIKV transmission (22). Furthermore, variations in sanitation and hygiene practices, as well as the effectiveness of vector control measures, could influence the prevalence of Chikungunya. Another aspect to consider is diagnostic capacity; differences in the accuracy and sensitivity of disease detection methods could lead to variations in reported prevalence. The difference in the seroprevalence observed between different regions is likely due to the extensive distribution and high population density of vectors, particularly in urban centers, which are factors that favor their occurrence. The prevalence of this pest was greater in the town, especially at internally displaced population sites, where various water containers, such as tyres, clay pots, barrels, plastic water tanks, flower vases, and old cars, are widely present as potential breeding grounds for Aedes mosquitoes (38). Additionally, the remoteness of certain areas may also play a role, as there are inequalities in the distribution of health facilities. These findings underscore the importance of considering the geographical context when implementing control and prevention strategies.

The current systematic review and meta-analysis demonstrated considerable variability in the seroprevalence of Chikungunya among occupations in Ethiopia. The highest seroprevalence of Chikungunya was found among farmers compared to individuals in other occupations (33). These findings are consistent with those of a study conducted in northwest Ethiopia by Ferede et al. (34). The variation may be associated with the degree of exposure to vectors transmitting CHIKV. Seropositivity for Chikungunya is greater in individuals who regularly move in forests, engage in agricultural activities, or have documented incidents of mosquito bites (6). Arboviruses typically circulate in forested areas through a sylvatic cycle involving primates as reservoir hosts (6). A study by Thiberville et al. (6), who reported that most seropositive individuals were engaged in farming activities, supported the higher prevalence of Chikungunya among farmers.

The prevalence of CHIKV in the reports included in this systematic review and meta-analysis was greater in the 31–40 years age group. Similar findings were reported from Tanzania (39). The higher infection rate in this age group may suggest that people in those age groups are active workers and exposed to the bites of the vector that transmits the disease. However, one study by Geleta et al. (17) reported a higher prevalence of Chikungunya in the 5–14 years age group and the lowest prevalence in the ≤5 years age group. This difference may be a result of the sample size difference, as the number of people aged 5–14 years who underwent diagnosis was almost double that of people aged ≥30 years.

According to the results of this systematic review and meta-analysis, education was associated with the seroprevalence of Chikungunya in Ethiopia. People who attended formal education were more strongly affected than people who did not attend formal education. This may be associated with educated people might be living in cities or towns where favorable environments for vectors, such as containers with water, are found. Educated people might have a more frequent migration history than non-educated people (6). Moreover, a higher prevalence of COVID-19 in educated individuals might be associated with better access to healthcare facilities as they live in cities where better health infrastructures are present, leading to increased detection and reporting of Chikungunya cases.

The results of these studies were inconsistent with those of sex. The prevalence of Chikungunya in males was greater than that in females according to Asebe et al. (35) and Ferede et al. (34). These findings are in line with other studies revealing that men are more susceptible than women are (40, 41). This may affirm the argument that males face a greater likelihood of encountering mosquito bites in the course of agricultural work or other comparable travel and occupational activities. In contrast, Endale et al. (33) reported that there were more CHIKV IgG+ females than males, indicating that females were more susceptible than males were. This opposite trend is supported by other seroprevalence studies (42, 43), in which females were more susceptible than males were. These conflicting reports highlight the necessity of further exploration of the associations between arbovirus infection and sex.

The results of subgroup analysis based on districts and test types were inconsistent across studies. The subgroup analysis, categorized by the type of tests conducted, revealed differences in study outcomes. Districts employing ELISA demonstrated significant variability among the studies compared to districts that utilized RT–qPCR, which exhibited a lack of variability among the studies, although this difference was not statistically significant. The overall heterogeneity was found to be high. These findings underscore the importance of accounting for the type of test in the analysis, as it appears to be a significant factor contributing to the observed heterogeneity. Further exploration of the sources of variability and careful consideration of the clinical implications are essential for a comprehensive interpretation of these results.

### 4.1 Study strengths and limitations

The strengths of this systematic review and meta-analysis are that the study included published data since the first detection of Chikungunya, and it is the first to report the pooled seroprevalence of Chikungunya in Ethiopia. This study has several limitations. The number of included studies was limited due to the restricted pool of available research, and the pooled prevalence may not accurately reflect the current reported rates. Almost all the included studies were cross-sectional. No molecular studies were carried out at the country level, except for RT–qPCR tests for confirmation. This limitation makes it challenging to predict circulating strains of the Chikungunya virus.

## 5 Conclusion and recommendations

In the current systematic review and meta-analysis, the pooled seroprevalence of Chikungunya was 24.0%. In addition, geographical location, occupation, age, sex, and education contributed to the variation in the Chikungunya seroprevalence. The subgroup analysis based on the study area and the type of tests performed revealed significant heterogeneity. The observed seroprevalence of Chikungunya reveals that the disease is remain persistent public health concerns in Ethiopia. Therefore, recognizing the significance of proactive operational readiness in mitigating the spread of infectious diseases during an outbreak is recommended. Furthermore, it is crucial for the Ministry of Health and other concerned bodies to emphasize collaboration and public awareness campaigns to better respond to similar outbreaks.

## Data Availability

The raw data supporting the conclusions of this article will be made available by the authors, without undue reservation.
